# Solution structure of the human signaling protein RACK1

**DOI:** 10.1186/1472-6807-10-15

**Published:** 2010-06-08

**Authors:** Kaliandra A Gonçalves, Julio C Borges, Julio C Silva, Priscila F Papa, Gustavo C Bressan, Iris L Torriani, Jörg Kobarg

**Affiliations:** 1 Laboratório Nacional de Biociências (LNBio), Centro de Pesquisa em Energia e Materiais (CNPEM), Campinas, SP, Brazil; 2 Instituto de Biologia, Departamento de Bioquímica, Universidade Estadual de Campinas - UNICAMP, Campinas, SP, Brazil; 3 Instituto de Química de São Carlos, Universidade de São Paulo, São Carlos, SP, Brazil; 4 Instituto de Física "Gleb Wataghin", Universidade Estadual de Campinas - UNICAMP, Campinas, SP, Brazil; 5 Laboratório Nacional de Luz Síncrotron (LNLS), Centro de Pesquisa em Energia e Materiais (CNPEM), Campinas, SP, Brazil; 6 Departamento de Bioquímica e Biologia Molecular Universidade Federal de Viçosa, Minas Gerais, Brazil

## Abstract

**Background:**

The adaptor protein RACK1 (receptor of activated kinase 1) was originally identified as an anchoring protein for protein kinase C. RACK1 is a 36 kDa protein, and is composed of seven WD repeats which mediate its protein-protein interactions. RACK1 is ubiquitously expressed and has been implicated in diverse cellular processes involving: protein translation regulation, neuropathological processes, cellular stress, and tissue development.

**Results:**

In this study we performed a biophysical analysis of human RACK1 with the aim of obtaining low resolution structural information. Small angle X-ray scattering (SAXS) experiments demonstrated that human RACK1 is globular and monomeric in solution and its low resolution structure is strikingly similar to that of an homology model previously calculated by us and to the crystallographic structure of RACK1 isoform A from *Arabidopsis thaliana*. Both sedimentation velocity and sedimentation equilibrium analytical ultracentrifugation techniques showed that RACK1 is predominantly a monomer of around 37 kDa in solution, but also presents small amounts of oligomeric species. Moreover, hydrodynamic data suggested that RACK1 has a slightly asymmetric shape. The interaction of RACK1 and Ki-1/57 was tested by sedimentation equilibrium. The results suggested that the association between RACK1 and Ki-1/57(122-413) follows a stoichiometry of 1:1. The binding constant (KB) observed for RACK1-Ki-1/57(122-413) interaction was of around (1.5 ± 0.2) × 10^6 ^M^-1 ^and resulted in a dissociation constant (KD) of (0.7 ± 0.1) × 10^-6 ^M. Moreover, the fluorescence data also suggests that the interaction may occur in a cooperative fashion.

**Conclusion:**

Our SAXS and analytical ultracentrifugation experiments indicated that RACK1 is predominantly a monomer in solution. RACK1 and Ki-1/57(122-413) interact strongly under the tested conditions.

## Background

The adaptor 36 kDa protein RACK1 (receptor of activated kinase 1) was originally identified as an anchoring protein for protein kinase C and contains seven WD repeats that mediate its protein-protein interactions [1,2]. It is also found to be up-regulated in human carcinomas and during tissue regeneration after ischemic renal injury [3,4]. Furthermore, RACK1 has been functionally implicated in the development of cardiac hypertrophy,[5] in the increase of focal adhesion[6] and regulation of cell adhesion [7]. Also, recent data suggest that RACK1 may affect gene expression through translation regulation and assembly activation of ribosomes [8-10]. Overall, these data suggest that RACK1 is a multi-purpose protein whose regulatory roles may impact various functional contexts.

It has been reported that RACK1 interacts with several molecules including β PKC β;[11] Src;[12] β integrins;[13] PDE4D5;[14] STAT1[15] and the regulatory protein Ki-1/57, [16,17] a cytoplasmic and nuclear protein initially identified in malignant cells from Hodgkin's lymphoma [18,19]. Recent studies showing its phosphorylation, arginine methylation and interaction with other regulatory proteins, suggest that the functional role of Ki-1/57 in human cells seems to be related to splicing regulation and other events in RNA metabolism [16,20,17,21].

RACK1 is a member of the family of β-propeller proteins and the first member of this protein family whose structure has been determined was the β-subunit of the mammalian heterotrimeric G protein [8,22]. Significant sequence similarity with the G protein β-subunit has led to the prediction that RACK1 may also adopt a seven-bladed β-sheet propeller [22,23]. This suggests the availability of multiple protein interaction surfaces. We previously modeled the three-dimensional structure of human RACK1[24] and the crystal structure of a RACK1 ortholog from *Arabidopsis thaliana *was recently elucidated with a resolution of 2.4 Å (PDB entry 3md0) [25].

Here, we analyzed the structural properties of human RACK1 in solution through a set of biophysical approaches. We showed that RACK1 is a predominantly monomeric protein in solution accordingly to data obtained from analytical ultracentrifugation (AUC) and small angle X ray scattering (SAXS) experiments. We restored the human RACK1 envelope from SAXS curves through *ab initio *approaches and compared it with previously obtained related structures. These results suggest a strong similarity between the structures of human RACK1 and RACK1 isoform A from *Arabidopsis thaliana*, in accordance with the high conservation between both amino acids sequences. The functional activity of RACK1 in solution was also accessed through its binding to Ki-1/57 protein. Fluorescence spectroscopy experiments after titration of Ki-1/57(122-413) in solution with RACK1 and sedimentation equilibrium experiments showed that this association is strong and might involve cooperative events.

## Results and Discussion

### Small Angle X-Ray Scattering results

**Figure 1:**
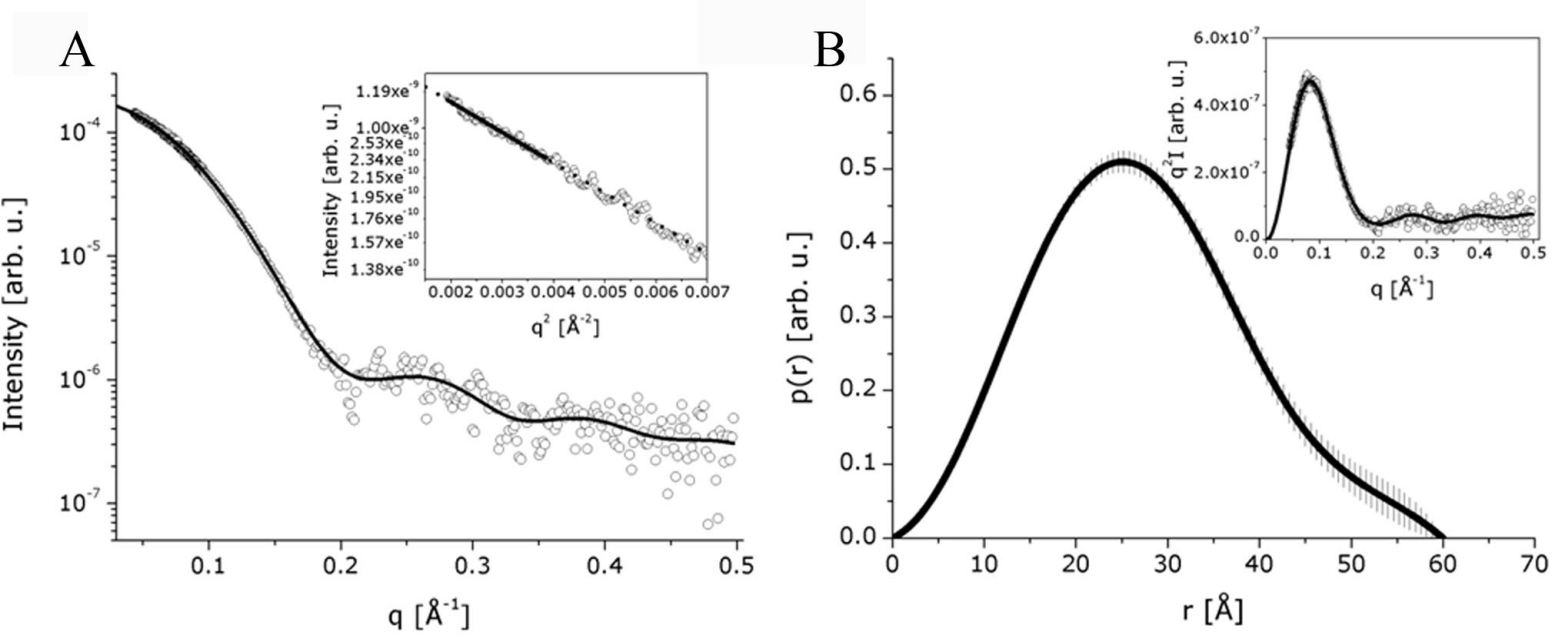
**Small Angle X-ray Scattering (SAXS) results**. (A) Experimental scattering curve of 6xHis-RACK1 (open circles) and the theoretical fitting (solid line) by using the program GNOM. *Inset*: Guinier Region and linear regression (solid line) for R_g _evaluation. (B) Pair distance distribution function p(r). *Inset*: Kratky plot.

Small-angle X-ray Scattering (SAXS) is an important technique to obtain shape information and dimensional parameters of biological macromolecules. Recently, the application of SAXS to predict low resolution protein structures gained even more importance with the advances in the X-ray sources and computational methods. Since efforts to crystallize the human RACK1 have been so far unsuccessful, an important step in the structural analysis of this protein is to perform X-ray scattering experiments to determine its conformation in solution. The obtained intensity SAXS curve, normalized by concentration, is presented in Figure 1A together with the regularization fit obtained from the indirect Fourier transform, which provided the pair distance distribution function p(r) as described in the "materials and methods" section. The resulting p(r) function is displayed in Figure 1B. In order to confirm the sample monodispersity, we inspected the Guinier [26] approximation in its validity region of the intensity curve. Guinier plots are displayed in the *inset *of Figure 1A. The linearity of this region confirmed its monodispersity. The radius of gyration (R_g_) of the human RACK1 in solution was (20.8 ± 0.8) Å, obtained using the Guinier approximation, and (20.6 ± 0.4) Å when calculated from the normalized second moment of the p(r) function. Both R_g _values are in very close agreement. The maximum dimension (D_max_) of the human RACK1 in solution provided by the p(r) function was 60 Å. These data suggest that the protein is globular and slightly oblate. The Kratky plot shown in the *inset *of Figure 1B indicates that the protein conformation is quite compact. Using a bovine serum albumin (BSA) solution as a standard, the MM estimated for human RACK1 from the SAXS data was ~40 kDa, which is very close to the value of 37 kDa predicted from the amino acid sequence of the recombinant protein. Based on these results we conclude that the human RACK1 protein is a monomer in solution.

### Amino acids sequence analysis and comparison of known structures of RACK1 with experimental SAXS data

**Figure 2:**
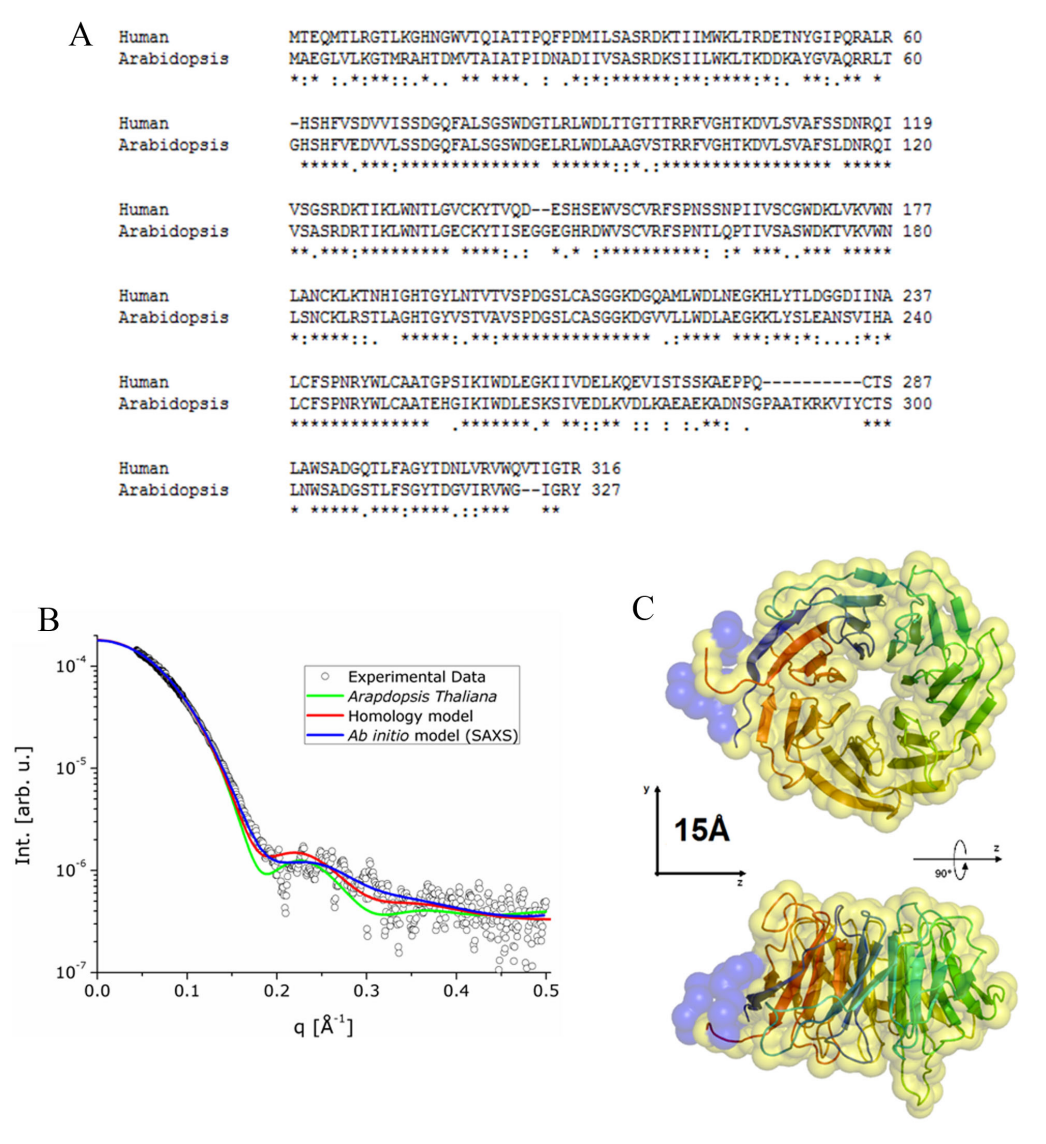
**Sequence alignment and *ab initio *model for human 6xHis-RACK1 protein in solution**. (A) Alignment between amino acid sequences of RACK1 from *Homo sapiens *(GI:83641897) versus RACK1A from *A. thaliana *(GI:30685669). The symbols: * showing identical residues, : residues with high similarity and residues with low similarity. (B) Experimental scattering curve of human 6xHis-RACK1 (open circles) and the calculated scattering intensity from the RACK1 from *A. thaliana *(solid green line) and *Homo sapiens *(solid red line) by using the program CRYSOL. The solid blue line represents the scattering intensity calculated from the *ab initio *model of 6xHis-RACK1. (C) Two orthogonal views of the homology model of human RACK1 (cartoon representation with secondary structure elements) superposed to the three dimensional low resolution *ab initio *model (semi-transparent yellow surface) of the human RACK1, obtained from processing the SAXS data.

Human RACK1 has significant amino acid sequence similarity to RACK1 isoform A from *Arabidopsis thaliana *with 66% amino acid sequence identity and 78% amino acid sequence similarity (Figure 2A). This comparison suggests that both proteins may have a very similar three dimensional structures. The alignment of both amino acid sequences shows that the main difference between the two proteins is located in their C-terminal region. RACK1 orthologs have also been discovered in unicellular eukaryotes, such as *Chlamydymonas,*[27] and are highly conserved in plants [28]. The high degree of sequence conservation likely reflects a high degree of functional conservation of the proteins in these species. Although mutations are acquired through evolution the preservation of the protein structure is usually higher than the conservation of the amino acid sequence, since the maintenance of the structure is pivotal for the proteins function [29].

The crystallographic structure of the RACK1 isoform A from *Arabdopsis thaliana *was recently solved and deposited in the Protein Data Bank under the code 3dm0 [25]. Furthermore, the homology model of the human RACK1 was previously published, [24] allowing to calculate the scattering intensity curve from the atomic coordinates of these two structures as described in the "material and methods" section. We compared these calculated curves with the experimental data obtained for the human RACK1 in solution. The results are shown in Figure 2B. Both calculated curves compare quite well with the experimental data, which indicates that the human RACK1 may have a very similar structure compared to both the homology model and the RACK1 isoform A from *Arabdopsis thaliana*, although the calculated intensity for the homology model (red curve) seems to be nearer to the experimental data for human RACK1.

### Low resolution models obtained from SAXS data

One of the great advantages of the SAXS technique is that it offers the possibility of restoring the three dimensional molecular envelope from the experimental data. This modeling can be improved when portions of the structure are already known, solving the possible ambiguities resulting from the calculation. This seems to be the case of the human RACK1, since both a homology model of human RACK1 as well as the crystal structure of RACK1 from *Arabidopsis thaliana*, are available. Since we studied the protein fused to a 6xHis tag at the N-terminal and we already observed that the homology modeled human RACK1 structure provided a calculated SAXS curve in agreement with the experimental SAXS data, we restored the human RACK1 molecular envelope using the method of addition of missing loops. Fixing the known part of the human homology model, we built dummy residues (DR) chains in the position attached to the proper amino acids in the N-terminal region of the structure. The number of DRs was known from the amino acid sequence. Both programs CHADD and GLOOPY provided very similar SAXS data based models for the human RACK1.

The resulting model is presented in Figure 2C (yellow spheres) superimposed to the *ab initio *homology model (ribbons representation). For the correct alignment of the two models, we used the program SUPCOMB [30,31]. To show the region of the *ab initio *model where the (6xHis tag) was attached to the protein, the dummy elements are shown as blue spheres. The intensity curve calculated from the model (derived from SAXS data) is displayed in the Figure 2B (black curve), showing, as expected, an even better fit to the experimental data. These results give a strong indication of the similarity of the structure of human RACK1 and RACK1 isoform A from *Arabdopsis thaliana *and confirms the structure predicted by the homology model of human of RACK1.

### Analytical ultracentrifugation experiments

**Figure 3:**
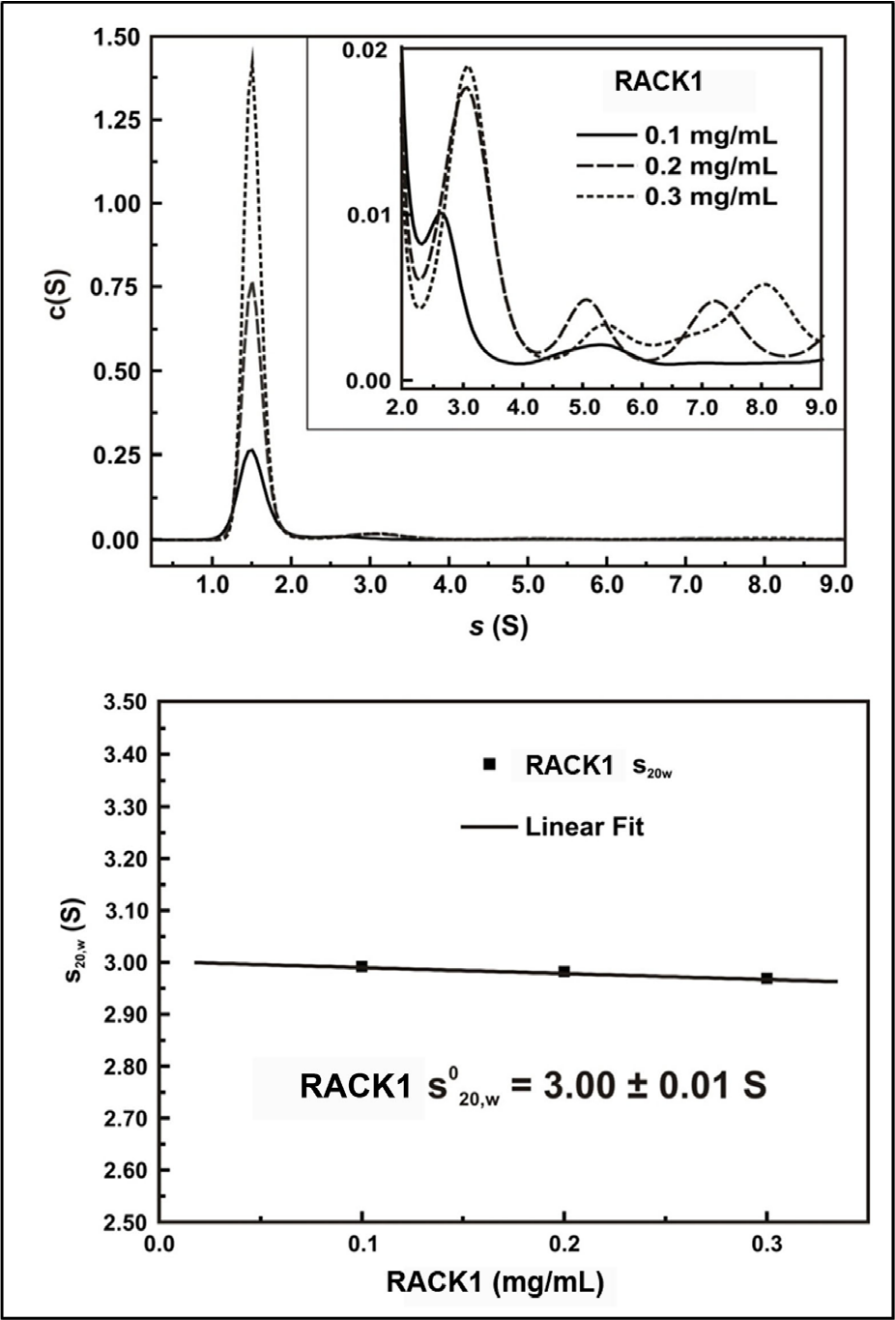
**RACK1 sedimentation velocity experiments**. (A) Sedimentation velocity experiments were carried out at 4°C, 40,000 rpm (AN-60Ti rotor), and with scan data acquisition at 272 nm. Data were fitted using SedFit software (Version 11.8). Figure displays the c(S) fitting for RACK1 at concentrations of 0.1, 0.2 and 0.3 mg/mL. The c(S) curves suggest that RACK1 present a predominant specie and three or four others species with higher sedimentation coefficients (*inset*) (B) Plots of s_20,ω _(of the predominant specie) versus protein concentration fitted by linear regression to calculate the s^0 ^_20;w: _3.00 ± 0.01 Svedberg.

Aiming to gain further informations on the structural parameters of human RACK1 in solution through its hydrodynamic properties, we performed sedimentation velocity experiments in an analytical ultracentrifuge (AUC). All experiments were performed at 3 protein concentrations, 4 °C, and the data were analyzed by the SedFit software. Figure 3A show the continuous c(S) distribution which suggests that the protein present one predominant species and, at least, 3 other species in solution (Figure 3A, *inset*). RACK1 as monomer should be the main particle in solution (~90% of the total mass) as observed on the area under the c(S) curve ratio. RACK1 as dimer (~6%), tetramer (~2%) and a hexamer or octomer (~2%), could be also observed in the c(S) curve (Figure 3A, *inset*). Moreover, these data are in accordance which previous observations that RACK1 tends to oligomerize and/or aggregate in multi-oligomer species depending on the storage conditions, such as ionic strength, protein concentration and temperature [24]. This property also forced us to include an ultracentrifugation step before subjecting our samples to SAXS data acquisition in order to solve problems with polydispersity (for details see Materials and Methods section).

The predominant monomeric particle in solution observed for RACK1 sedimentation velocity experiments provided a s^0^_20,w _of 3.00 ± 0.01 S as presented in Figure 3B. We estimated the diffusion coefficient by dynamic light scattering at 4 °C and corrected it to standard condition (D_20,w_, Materials and Methods). Both s^0^_20,w _and D_20,w _were used to calculate the RACK1 MM through the s/D ratio (Equation 1) and the result suggest that the predominant particle in solution was a monomer of around 37 kDa (Table 1). In spite of differences in the buffers used in SAXS experiments (containing 20% of glycerol and 150mM of NaCl) and AUC experiments (3% of glycerol and 500 mM of NaCl), the results of both techniques suggest that RACK1 is a monomer in solution. Indeed, the polydispersity observed in the AUC experiments may be artifactual and may have been caused by the lower glycerol content in the AUC buffer, the larger incubation time in this experiment (in comparison with the SAXS experiments) and finally also the relatively higher protein concentration used here. All of these conditions may promote the observed protein aggregation in the AUC experiments.

**Table 1:** RACK1 hydrodynamic properties.

RACK1 Hydrodynamic property	Predicted for a sphere	DLS*	AUC	HydroPro of homology model	SAXS
*M *(kDa)	37.2	36.4 ± 0.8^&^	36.4 ± 0.8^&^	-	40
*s^0^20,w *(S)	4.01	-	3.00 ± 0.01	3.16 ± 0.02	-
*D20,w *(10^-7^cm^2^/seg)	9.6	7.7 ± 0.2	-	8.0 ± 0.1	-
*Dmax *(Å)	-	-	-	63 ± 1	60
*Rg *(Å)	-	-	-	20.0 ± 0.1	20.8 ± 0.8^$^
					20.6 ± 0.4^#^

^&^from s/D ratio (Equation 1)

$: from Guinier approximation

#: from the normalized second moment of the p(r) function

* Dynamic light scattering

Table 1 summarizes the RACK1 hydrodynamic properties calculated for a sphere of 37 kDa (RACK1's MM) and the experimental data determined by dynamic light scattering and sedimentation velocity. We also used the RACK1 homology model previously calculated [24] to obtain its hydrodynamic properties using HydroPro software (see Material and Methods) [32]. Both D_20,w _and s^0^_20,w _determined experimentally were in accordance to the hydrodynamic properties calculated for the homology model, suggesting that it is a reasonable model for RACK1 in solution. All hydrodynamic data, together with the frictional ratio calculated by either SedFit software (~1.4) or estimated from the ratio of s_sphere _to s^0^_20,w _(~1.3), suggest that RACK1 is a slightly asymmetric or distorted particle.

**Figure 4:**
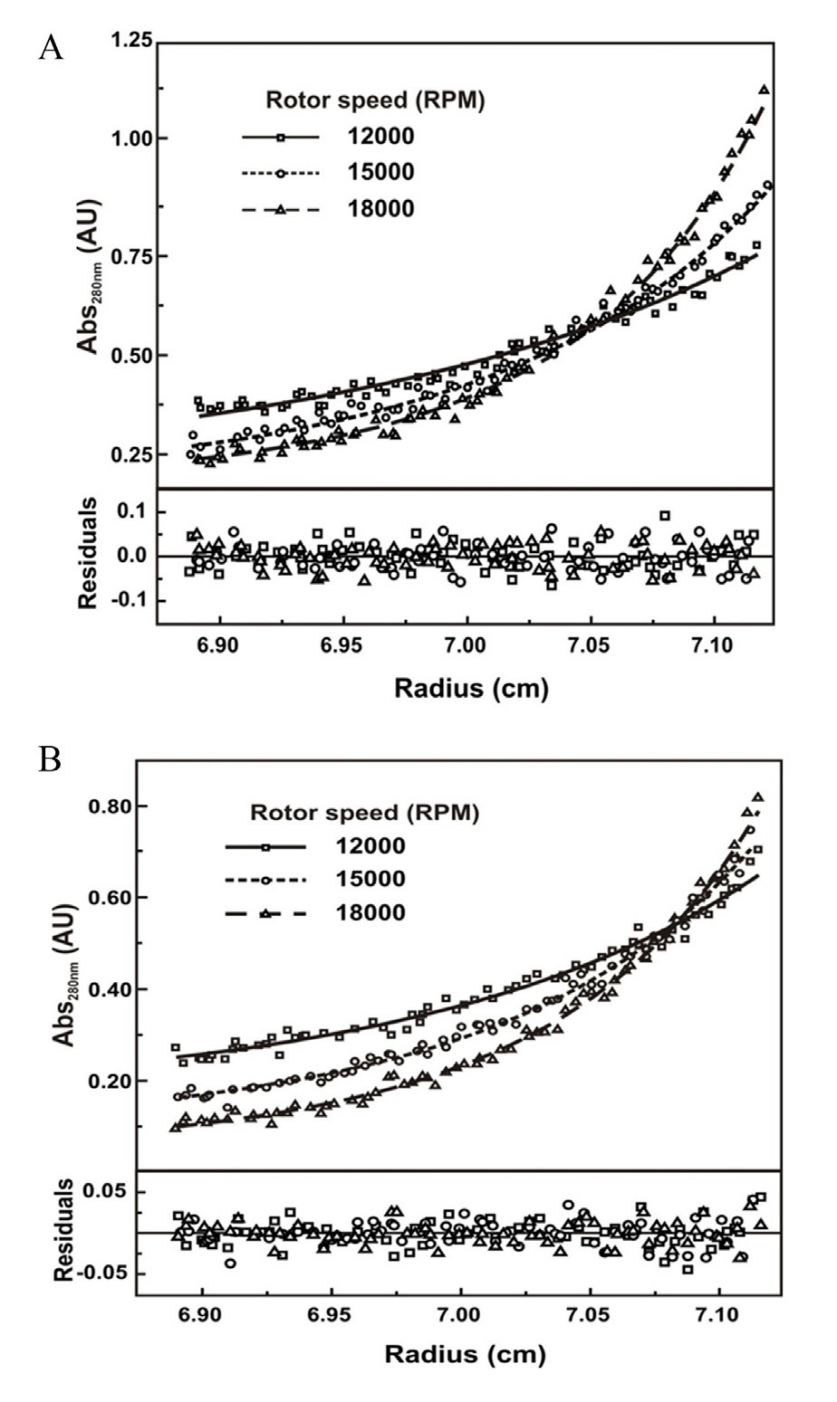
**RACK1 Sedimentation equilibrium experiments**. (A) The figure shows the best fits of experimental data for 300 μg/mL of RACK1 (4 °C) at 12000, 15000, and 18000 rpm with the self-association methods (SedPhat program - Material and Methods). The random distribution of the residuals (bottom panel) indicates that the fit is satisfactory. Sedimentation equilibrium data of RACK1 agree with a monomer structure with 37.2 kDa in equilibrium with of dimers and tetramers (see Results and Discussion for details). (B) Sedimentation equilibrium interaction between RACK1 and KI-1/57(122-413) was tested at 300 μg/mL of both proteins at 12,000, 15,000, and 18,000 rpm and 4 °C. The data were fitted by self-association method (SedPhat program - Material and Methods) and resulted in random distribution of the residuals (bottom panel) which indicates that the fit is satisfactory. The fitting data suggest that RACK1 and Ki-1/57(122-413) interacts with an affinity constant of (1.5 ± 0.2) × 10^6 ^M^-1^.

Equilibrium sedimentation is an analytical ultracentrifugation method based on the equilibrium between the centrifugal and diffusional forces. It is a thermodynamic technique that allows to obtain the MM of a particle independently of its molecular shape and to perform protein association studies [33]. RACK1 equilibrium sedimentation experimental data fitted with a model of a single specie system (variable MM) and resulted in a MM of around 46 kDa (data not shown), which suggested that human RACK1 solutions contained monomers of 37 kDa and oligomers, as also observed in sedimentation velocity experiments. Thus, we fitted the equilibrium sedimentation data using several models of self-associating systems. Considering that the sedimentation velocity experiments presented above suggested the presence of a monomer-dimer-tetramer system (Equation 2), we used a model of self-association to fit the sedimentation equilibrium data, using the SedPhat software. The presence of the hexamer or octomer, however was not considered for this fitting analysis, because of their low molar concentration in the human RACK1 solution used. Figure 4A (upper panel) presents RACK1 at 0.3 mg/mL in equilibrium at 3 speeds in which the data were fitted with the Equation 3. For this, we fixed the monomer MM at 37.2 kDa. The lower panel in Figure 4A, shows a residuals fitting dispersed around zero, which points to a good fitting. As expected, human RACK1 formed dimers and tetramers with KB_M-D _of (2 ± 0.2) ± 10^5 ^M^-1 ^and KB_D-T _of (6 ± 2) × 10^5 ^M^-1^, respectively. Dissociation constant (KD) for monomer-dimer (KD_M-D_) and dimer-tetramer (KD_D-T_) equilibrium where of 51 ± 2 μM and 17 ± 9 μM, respectively.

Thus, our findings that RACK1 is predominantly a monomer in solution seem at first to contrast with some data found in the literature, that relate that human RACK1 can engage in dimerization interaction in *in vitro *pull-down assays and *in vivo*, using cross-linker experiments [34]. A more recent study however, demonstrates that such a RACK1 dimerization *in vivo *may depend crucially on its previous phosphorylation, while dephosphorylation of RACK1 causes the disintegration of the homodimeric complex [35] Altogether, these findings are in good agreement with our results, since our bacterially expressed RACK1 is not phosphorylated, whereas RACK1 in human cells is likely to be phosphorylated and regulated by phosphorylation. Therefore, the dephosphorylated RACK1 should present low KB while the phophorylated RACK1 may be able to dimerize with higher KB. However, we cannot exclude that the relative low KB_M-D _and K_D-T _described above may be an artifact induced by the experimental conditions tested.

### RACK1 binding activity in solution

We also used sedimentation equilibrium methods to assess how functional RACK1 protein was in our experimental conditions. Previously, we described the interaction of RACK1 with the regulatory protein Ki-1/57 and analyzed their interaction through spectroscopic methods [17,24]. We then used here a truncated form of Ki-1/57 encompassing the amino acids 122-413 to obtain further thermodynamic details of this association. We analyzed the absorbance data by the SedPhat program as multi-speed equilibrium data and the results are presented in Figure 4B by using the Equation 3 presented above for a hetero-association system with a stoichiometry between RACK1 and Ki-1/57(122-413) of 1:1 (Equation 4). The lower panel (Figure 4B) shows the residuals fitting randomly dispersed around zero, which points to a good data fitting. The KB observed for RACK1-Ki-1/57(122-413) interaction was of around (1.5 ± 0.2) × 10^6 ^M^-1 ^that resulted in a KD of 0.7 ± 0.1 μM. This data suggest that the equilibrium interaction between these two proteins is strong in the tested conditions. The KB for RACK1-Ki-1/57(122-413) interaction is 7.5 times higher than RACK1 KB_M-D _suggesting that, in the protein concentration assayed, around 91% of the RACK1 is in the hetero-dimeric complex and that the concentration of RACK1 as a homo-dimer must be lower than 0.01 μM and could be neglected from this analysis. This highly productive association may explain why around 54% of clones restored from our yeast two-hybrid screenings using Ki-1/57 as bait represented the RACK1 protein [16].

**Figure 5:**
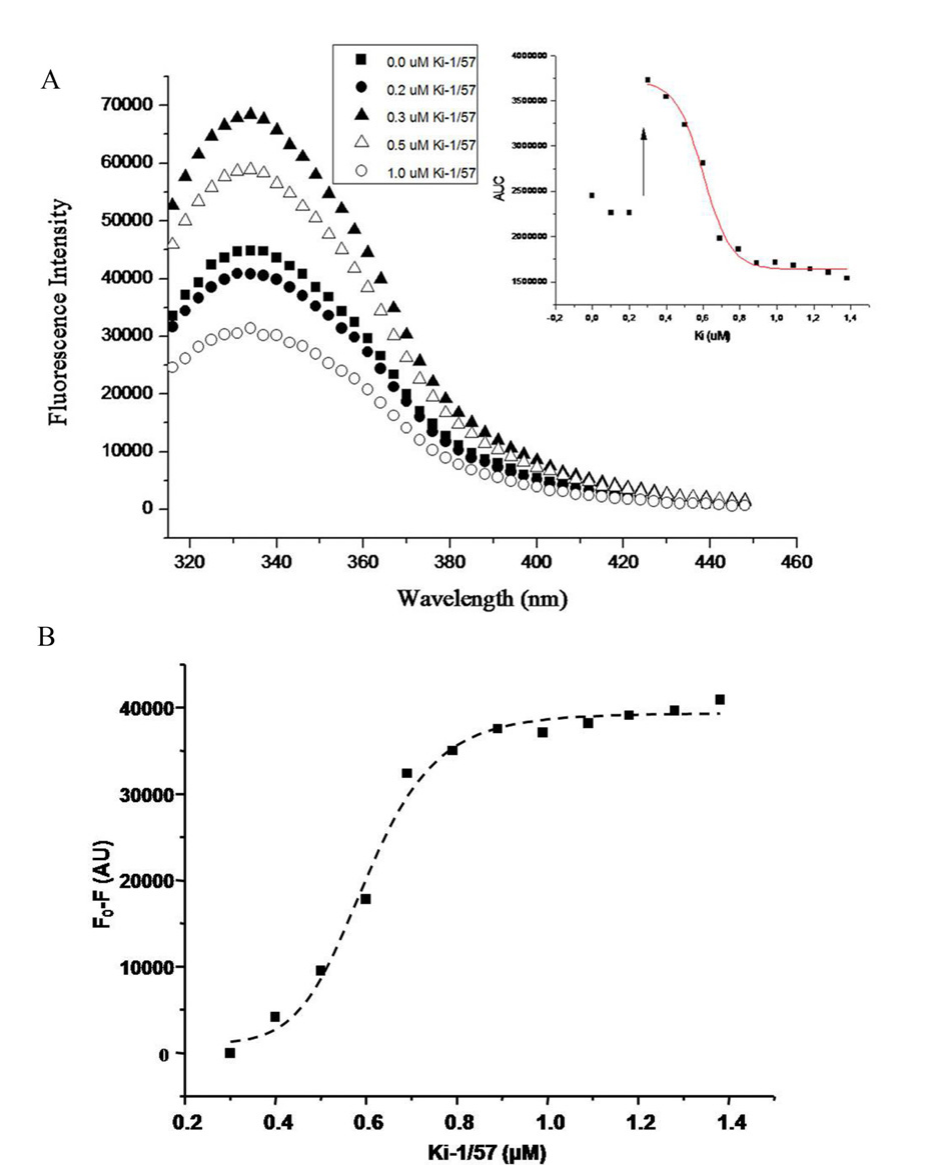
**Titration of Ki-1/57(122-413) into RACK1 solution monitored by fluorescence**. (A) Fluorescence emission spectra titration of purified 6xHis RACK1 protein (1 μM) with titration of Ki-1/57 at 0.1 μM (black, filled square), 0.2 μM (black, filled circle), 0.3 μM (black, filled triangle), 0.5 μM (open, white triangle) and 1 μM (open, white circle). The excitation wavelength was 295 nm and spectra were background corrected. *Inset*: Area under curve (AUC) of RACK1 fluorescence emission spectrum in function of Ki-1/57(122-413) concentration. The arrow indicates the increase on the RACK1 fluorescence signal at Ki-1/57(122-413) 0.3 uM. (**B**) The titration curve was represented as difference of fluorescence intensity at 334 nm θ (black, filled square) as function of Ki-1/57(122-413) concentration in the range from 0.3 μM and 1.4 μM and fitted with the modified Hill equation (Equation 5). The dashed line represents the fitting curve. The KD between RACK1 and Ki-1/57(122-413) was of around 0.60 μM which corresponds to the dissociation constant of RACK1\Ki-1/57(122-413) observed in sedimentation equilibrium experiments. The number of cooperative sites n was 8 ± 1.

Titration of Ki-1/57(122-413) to RACK1 solution was carried out at 25 °C in 20 mM Tris-HCl (pH 7.5) and followed by intrinsic fluorescence emission. However, we did not observe any induced changes in the maximum fluorescence emission wavelength upon Ki-1/57(122-413) titration (Figure 5A). On the other hand, the titration curve presented two well defined regions (Figure 5, *inset*) suggesting that the RACK1-Ki-1/57(122-413) interaction involves, at least, two effects: up to 0.2 μM of Ki-1/57(122-413), it is possible to observe an initial fluorescence decrease probably due to fluorescence quenching. At 0.3 μM of Ki-1/57(122-413), the emission fluorescence increased nearly twice suggesting that RACK1 and/or Ki-1/57(122-413) may undergo a conformational change upon Ki-1/57(122-413) binding (Figure 5, *inset*). We cannot explain this result, but we speculate that this intriguing effect may be a result of Ki-1/57(122-413) binding to the human RACK1 surface where at least part of the Trp residues seem to be exposed as our previous homology model of RACK1 suggested [24]. These Trp residues may be already quenched by the solvent and RACK1 association with Ki-1/57(122-413) could lead the tryptophans to a conformation able to show an increment in the total fluorescence intensity emission.

At concentrations higher than 0.3 μM the Ki-1/57 titration induced RACK1 fluorescence emission quenching which follows a sigmoidal curve (Figure 5, *inset*), that represents another effect due to protein association. Figure 5B represents the difference of fluorescence intensity at 334 nm (F_0 _- F = θ) as function of Ki-1/57 concentration range from 0.3 μM up to 1.4 μM. Adjusting this titration curve with a modified Hill equation (Equation 5) we could estimate the KD for the interaction between Ki-1/57 and RACK1 as about 0.60 μM and the number of cooperative sites n as 8 ± 1. This points to an apparent cooperativity of the interaction, since the stoichiometry determined by sedimentation equilibrium was 1:1. It is also interesting to note that this KD corresponds to the KD of RACK1\Ki-1/57(122-413) observed in sedimentation equilibrium experiments. These data emphasize that the interaction between human RACK1 and Ki-1/57 was strong in the experimental conditions tested.

The human RACK1 protein contains thirteen tryptophan residues, which yield strong signal fluorescence emission intensities but also increase the complexity of fluorescence emission spectra. Despite the high number of Trp residues in the RACK1 structure, the homology model developed for RACK1 suggests the presence of, at least, two kinds of Tryptophans on RACK1: 1) the exposed Trps on the RACK1 surface and 2) the buried or partially buried Trps [24]. Thus, these different classes of Trps may suffer distinct effects upon Ki-1/57(122-413) binding: while some of them may be more protected, others may be rather quenched. However, we cannot exclude the participation of the 3 Trps on the Ki-1/57(122-413) recombinant protein. Ki-1/57(122-413) has been described as an intrinsically unfolded protein [36] and therefore it could bind to RACK1 by several contact points, possibly inducing conformational changes on either protein. The sigmoidal titration curve observed may be explained by possible cooperative effects in the binding of the two proteins. However, more detailed emission fluorescence or other spectroscopic experiments are required to address this newly raised issue.

## Conclusion

Human RACK1 has significant amino acid sequence and structurally similarity to RACK1 isoform A from *Arabidopsis thaliana*. Analytical ultracentrifugation experiments showed that human RACK1 has similar hydrodynamic properties to that predicted to homology model previously developed. Moreover, the SAXS data demonstrated that the human RACK1 in solution may have the same or very similar molecular envelope as that of the RACK1 isoform A from *Arabidopsis thaliana*. Both techniques also showed that the homology model previously proposed by shows a good agreement with the experimental data presented here [24].

The hydrodynamic properties of the human RACK1 were established by both sedimentation velocity and dynamic light scattering. The samples presented an apparent aggregation in the hydrodynamic experiment and they showed polydisperse behavior. Although the equilibrium sedimentation analytical ultracentrifugation results indicated a tendency of the protein for di- and tetramerization, the data showed that human RACK1 is predominantly a monomer in solution (over 90%). SAXS data also confirmed the human RACK1 monomeric conformation in solution, although we previously adjusted the glycerol content in the buffer and centrifuged the sample to assure its monodispersity. This condition was checked by DLS before the SAXS experiments (data not shown).

The interaction of RACK1 with the Ki-1/57(122-413) is strong and follows the stoichiometry of 1:1 as observed by sedimentation equilibrium experiments. RACK1 is a Trp-rich protein and its fluorescence emission spectrum presents a maximum of around 334 nm suggesting that some of these Trp are exposed to the solvent. The interaction of RACK1 with the Ki-1/57(122-413) monitored by fluorescence emission spectroscopy agreed with the interaction strength observed in the sedimentation equilibrium experiments. Furthermore, the titration curve resembles a sigmoidal curve suggesting a cooperative effect in the interaction of RACK1 with the Ki-1/57(122-413). Considering that Ki-1/57(122-413) is an intrinsically unfolded protein,[36] its interaction with RACK1 may involve several binding sites and seems to induced conformational changes upon binding.

## Methods

### Plasmid constructions

Cloning of the cDNA coding for RACK1 full-length into the pET-28a plasmid (Novagen/EMD Biosciences, San Diego, CA) was performed as described previously [16]. The recombinant vector pET-28a-RACK1 expresses a construct containing 24 additional amino acids at the N-terminal of RACK1. These extra residues encopass the 6 × His-tag and other poly-linker encoded amino acids. cDNA encoding human Ki-1/57 protein fragments was amplified by PCR and then directionally cloned into the bacterial expression pPROEx (Invitrogen, Carlsbad, USA), as previously described [36].

### Expression and purification of recombinant proteins

BL21(Δ SlyD) strain (Invitrogen) was transformed with recombinant pET28a- RACK1 vector and grown in LB medium with 30 ug/ml of kanamycin. When reaching the logarithmic growth phase the recombinant bacteria were induced for protein production with 1.7 mM of isopropyl-b-D-thiogalactoside (IPTG) at 30 °C for 4 h. After harvesting (6000 × *g*, 10 min), the bacterial cells were resuspended and incubated for 40 min at 4 °C in lysis buffer: 50 mM Tris-HCl (pH 7.5), 150 mM NaCl, 20% glycerol, 1 mg/ml lysozyme, 1 mM phenylmethylsulfonyl fluoride, and 0.05 mg/ml DNase. Additional pellet disruption was performed by 10 cycles of sonication in an ice bath, followed by centrifugation at 18 000 × *g*, 4 °C for 30 min. The obtained supernatant was loaded onto a HiTrap Chelating HP column (Ge Healthcare) and eluted by a gradient of 0-400 mM imidazole. The obtained Ni^2+^-affinity purified fractions were pooled and dialyzed against the buffer: 50 mM Tris-HCl (pH 7.5), 150 mM NaCl, 20% glycerol.

To obtain protein preparations free of degradation and up to 95% of purity, Ki-1/57(122-413) was purified in two steps as follows as described previously [36]. The concentration of the recombinant protein was spectroscopically determined using the calculated extinction coefficient for the denatured proteins [37].

### SAXS experiments

The SAXS experiments were performed at the D02A-SAXS2 beamline of the Laboratório Nacional de Luz Síncrotron (LNLS, Campinas, Brazil). Before the analysis, the samples of human RACK1 were centrifuged at 356,000 × g for 30 min at 18 °C to remove any possible aggregates. The composition of the buffer solution was adjusted to avoid aggregation, [20 mM Tris-HCl (pH 7.5), 150 mM NaCl, 20% glycerol]. Dynamic light scattering (DLS) was used to test the monodispersity of the solution. Measurements were performed with a monochromatic X-ray beam with a wavelength of λ = 1.488 Å and the X-ray scattering patterns were recorded using a two-dimensional position-sensitive MARCCD detector. The sample-to-detector distances were set at 506.8 mm and 1722.5 mm, corresponding to the scattering vector range of 0.01 < q < 0.50Å^-1 ^where q is the magnitude of the ***q***-vector defined by *q *= (4*π*/*λ*)sin*θ *(where *2θ *is the scattering angle). Three successive frames of 300 s each were recorded for each sample. The scattering data from the buffer were recorded before and after the measurement of each sample. The experiments were performed with two different sample concentrations: 1.95 and 2.54 mg/mL. The sample and buffer intensity curves were individually corrected for detector response and scaled by the incident beam integrated intensity and the sample absorption. Subsequently, the buffer scattering was subtracted from the corresponding sample scattering. The resulting curves were inspected for radiation-induced damage, but no such effect was observed. A 5 mg/ml BSA (66 kDa) solution in the same sample buffer was used as MM standard sample to estimate the MM of RACK1. This value was inferred from the ratio of the extrapolated values of the intensity at the origin, *I(0)*, from both sample and BSA solutions scattering [38,39].

The initial analyses of the SAXS data were performed following the standard procedures. The radius of gyration and the forward scattering intensity *I(0) *were obtained from the Guinier approximation [26,40,41] where we assumed that the intensity can be represented as *I*(*q*) = *I*(0)exp(-(*qR*_*g*_)^2^/3) at very small values of q (*qR*_*g *_< 1). Thereupon, the pair distance distribution function p(r) was calculated by indirect Fourier transform of the scattering intensity through a regularization method implemented in the program GNOM [42]. This p(r) function also provided the *I(0) *and *R*_*g *_values. These parameters proved to be in agreement with those obtained from the Guinier approximation. This function also allows determination of the maximum dimension of the protein in solution since *p(r > Dmax) = 0*.

We compared the experimental scattering curve from the human RACK1 protein with its calculated scattering curve from the homology model from the human RACK1 and with the calculated curve from the crystallographic structure of the RACK1 isoform A from *Arabdopsis thaliana*. The calculation of the scattering intensity from the atomic coordinates of the latter structures was performed using the program CRYSOL [43]. Since the sample we studied was in a solution with 20% glycerol, we calculated the electron density of the solvent for this condition (0.3497 electrons/Å^3^). The contribution of the other components of the buffer solution to the the electron density of the water (0.3344 electrons/Å^3^) was considered negligible, due to their low concentration.

### *Ab initio *modeling from the SAXS data

The *ab initio *models of the human RACK1 in solution were calculated from the SAXS data using the homology model previously obtained. We used a modeling method that is an extension of the original Dummy-Residues (DR) approach [31]. It can be used when parts of the protein are known and the location of the interface of the known and missing parts is also known [44]. Here, as we studied the protein fused to a 6xHis tag at the N-terminal, the homology model of the human RACK1 is used as the known part. The 6xHis tag is represented as a chain of DRs attached to the proper residues (known from the sequence) in this structure. We performed random modification in this chain of DRs using the simulated annealing method for global minimization of the scoring function to provide the best fit to the experimental scattering curve. This method is implemented in two programs: [44] CHADD and GLOOPY. Both programs are similar but the second one takes into account the primary structure (amino acid sequence) of the protein.

### Analytical ultracentrifugation

Sedimentation velocity and equilibrium sedimentation experiments were performed using a Beckman Optima XL-A analytical ultracentrifuge and analyzed as previously described [45]. Briefly, sedimentation velocity experiments were carried out at 4°C and 40,000 rpm (AN-60Ti rotor) in buffer Tris-HCl 20 mM (pH 7.5), NaCl 500 mM and Glicerol 3% (0.404 M). Human RACK1 was tested at 0.1, 0.2 and 0.3 mg/ml and absorbance data acquisition was at 272 nm. The fitting of absorbance *versus *cell radius data was done using SedFit (Version 11.8), which solves the Lamm equation in order to discriminate the spreading of the sedimentation boundary from diffusion [46,47]. The maximum of peaks of the c(S) curves gave the apparent sedimentation coefficient (*s*). The frictional ratio (*f*/*f*_0_) was the regularization parameter in fitting routine. The relative amount of each particle in c(S) curves was estimated by the integral of the area under curve of each peak in the c(S) curve using SedFit software (Version 11.8) and OriginPro^® ^(Version 8). The software Sednterp http://www.jphilo.mailway.com/download.htm was used to estimate RACK1 partial specific volume at 20°C (*Vbar*= 0.7288 mL/g), buffer density at 4 °C (*ρ *= 1.02976 g/mL), buffer viscosity at 4 °C (*η*= 0.017934 poise), and also the *s*_*sphere *_and the *D*_*sphere *_for a globular protein of about 37.2 kDa (Table 1). This software was also used to estimate the standard sedimentation coefficient (*s*_*20,w*_) for each protein concentration in order to estimate the *s*_*20,w *_at 0 mg/mL of protein concentration (*s*^*0*^_*20,w*_) by linear extrapolation [45]. This procedure minimizes interferences caused by temperature, viscosity and molecular crowd [48]. The diffusion coefficient parameter, *D*, was obtained from dynamic light scattering with a DynaPro-MS/X device (Protein Solutions) at 4°C. The D_20,w _was calculated as described [49] in similar way that described above to *s*_*20,w*_. The MM can be estimated from the ratio of the *s *to *D *as indicated by the equation 1:(1)

where *R *is the gas constant, *T *is the absolute temperature.

The HydroPro software [32] was applied to estimate the standard diffusion coefficient (*D*_*20,w*_), standard sedimentation coefficient (*s*_20,w_) and hydrodynamic maximum distance (*D*_*max,h*_) from the RACK1 model previously developed [24] in order to compare to the experimentally hydrodynamic data.

Sedimentation equilibrium experiments were carried out at 4°C and at three speeds as follow: 12,000, 15,000 and 18,000 rpm (AN-60Ti rotor). Scan data acquisitions were taken at 280 nm after 12 h of centrifugation at each speed, in which the equilibrium was checked by subsequent scans. Sedimentation equilibrium analysis involved fitting of absorbance *versus *cell radius data using nonlinear regression routine implemented by SedPhap software (Version 6.5). The Self-Association method was used to analyze the sedimentation equilibrium experiments using several models of association for RACK1. As observed in the sedimentation velocity experiments, RACK1 tends to oligomerize, thus the self-association model described by the equation 2 applied, where KB_M-D _and KB_D-T _are the binding constant of monomer-dimer and dimer-tetramer, respectively (see Results and Discussion section for details).(2)

Distribution of the protein along the cell, obtained in the equilibrium sedimentation experiments, was fitted with the following equation for associating system.(3)

where *C *is the protein concentration at radial position *r*, *C*_*mononer,r0 *_is the protein concentration at radial position *r*_*0 *_(initial radial position), *ω *is the centrifugal angular velocity, KB is the binding constant and *n *is the stoichiometry. Equation 3 can be developed for equilibrium systems with more species.

Interaction between RACK1 (37.2 kDa) and the C-terminal of Ki-1/57(122-413) (37.3 kDa) [36] was also tested by equilibrium sedimentation. Both proteins were prepared in the buffer Tris-HCl 20 mM (pH 7.5), NaCl 500 mM and Glicerol 3% (0.404 M) at 300 ug/mL (~8.0 μM) and submitted to centrifugation in the same conditions presented above. SedPhat software (Version 6.5) [50] was used to fitting the hetero-association model following the Equation 4 where KB is the binding constant between RACK1 and the C-terminal of Ki-1/57(122-413). Again, Equation 3 was used for fitting protein distribution along the cell.(4)

The software Sednterp was used to estimate the molar absorbance of the folded protein at 280 nm for RACK1 (80.940 mol/L^-1^.cm^-1^) and the C-terminal of Ki-1/57(264-413) (32.890 mol/L^-1^.cm^-1^). These parameters were used in SedPhat software for hetero-association fitting.

### Fluorescence spectroscopy

The experiments were performed with an Aminco BowmanR Series 2 (SLM-Aminco) spectrofluorimeter equipped with a 450W lamp. Experiments were carried out at 25 °C in 20 mM Tris-HCl (pH 7.5). The recombinant proteins were analyzed at concentration of 1 μM. The intrinsic Tryptophan fluorescence was investigated with an excitation wavelength of 295 nm using a spectral band pass of 4 nm for both excitation and emission. The titration curve of RACK1 and Ki-1/57 was used to fit with a modified Hill equation [51] to determine the KD of the interaction as described above:(5)

where F_0 _is the fluorescence intensity at 0.3 μM of Ki-1/57, F is the fluorescence intensity at a given Ki-1/57 concentration (in μM), θ is the difference of fluorescence intensity, θ_0 _is θ at 0.3 μM of Ki-1/57; θ_max _is θ at Ki-1/57 saturating concentration, and n is the number of cooperative sites. The software OriginPro^® ^(Version 8) was used to this fitting routine.

## Authors' contributions

KAG and JK conceived and designed the experiments, analyzed the data and wrote the manuscript. KAG performed or participated in all experiments. JCB participated in analytical ultracentrifugation, spectroscopic experiments and performed their respective data interpretation. PFP helped with protein expression and purification optimization. JCS performed SAXS experiments and interpreted them together with ICLT. GCB helped in the designing of experiments and performed protein expression and purification. JK supervised the project. All authors read and approved the final version of the manuscript.
